# Supplementary figures and images for: Structural, mechanistic, and physiological insights into phospholipase A-mediated membrane phospholipid degradation in Pseudomonas aeruginosa
Source: eLife. 2022 May 10;11:e72824. doi: 10.7554/eLife.72824 (PMC9132575; doi:10.7554/eLife.72824)

original

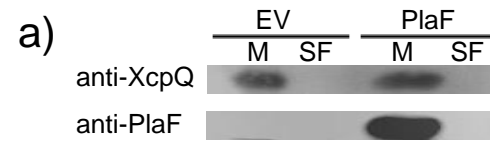

uncropped

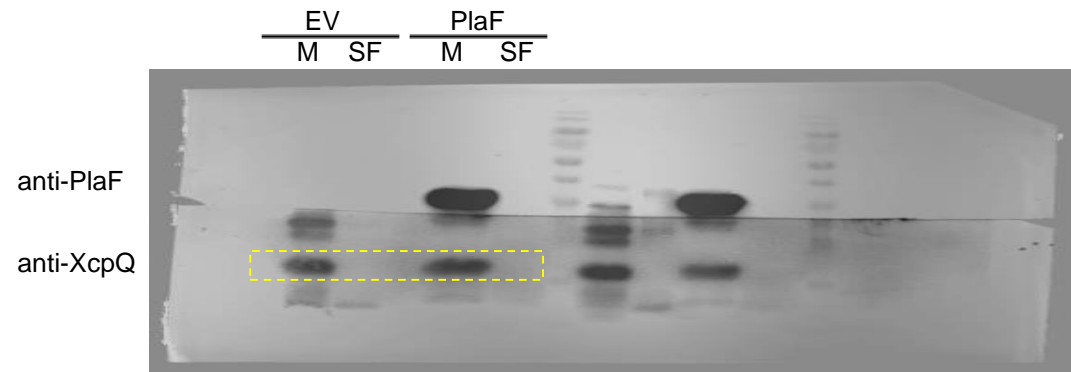

Figure 1a

Supplement: Figure 1—source data 1. [file elife-72824-fig1-data1.pdf]

original

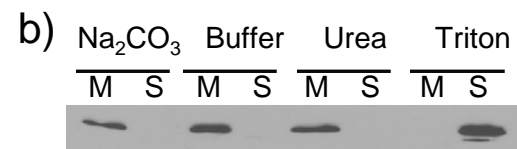

uncropped

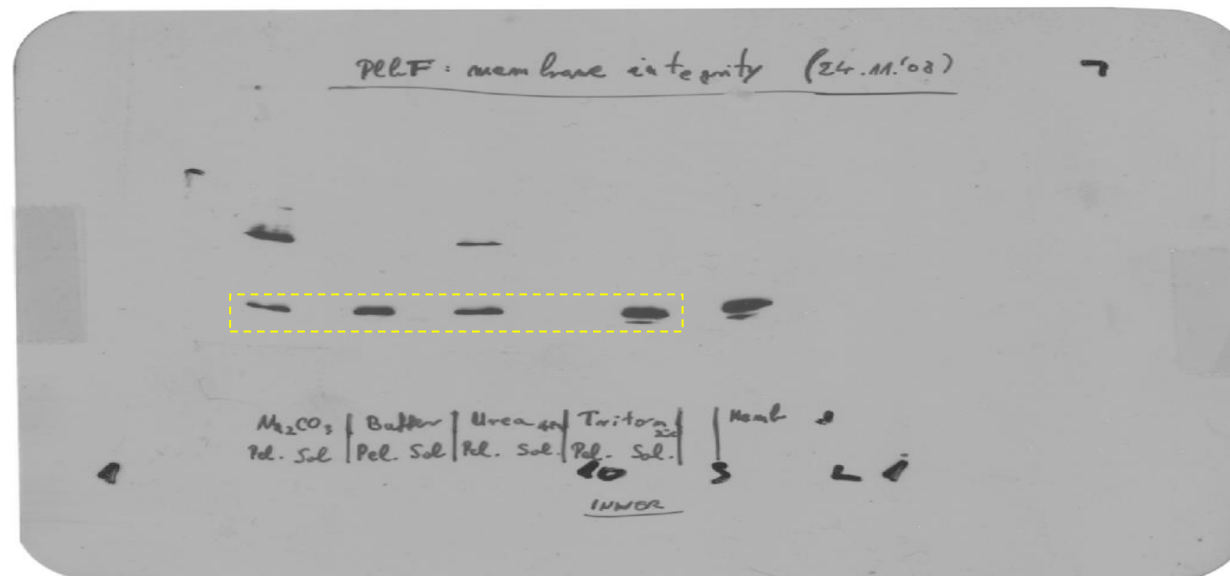

Figure 1b

Supplement: Figure 1—source data 2. [file elife-72824-fig1-data2.pdf]

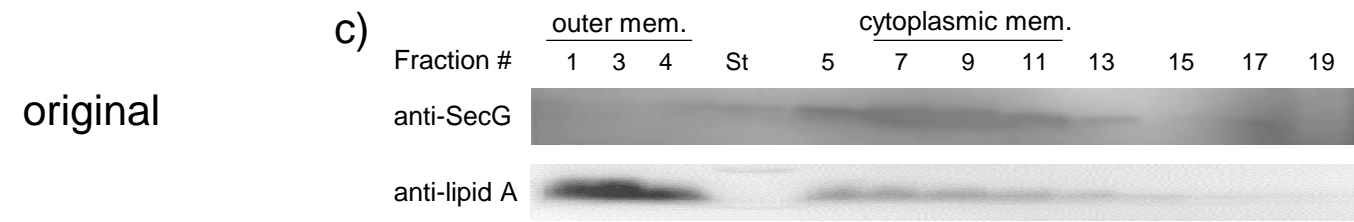

uncropped

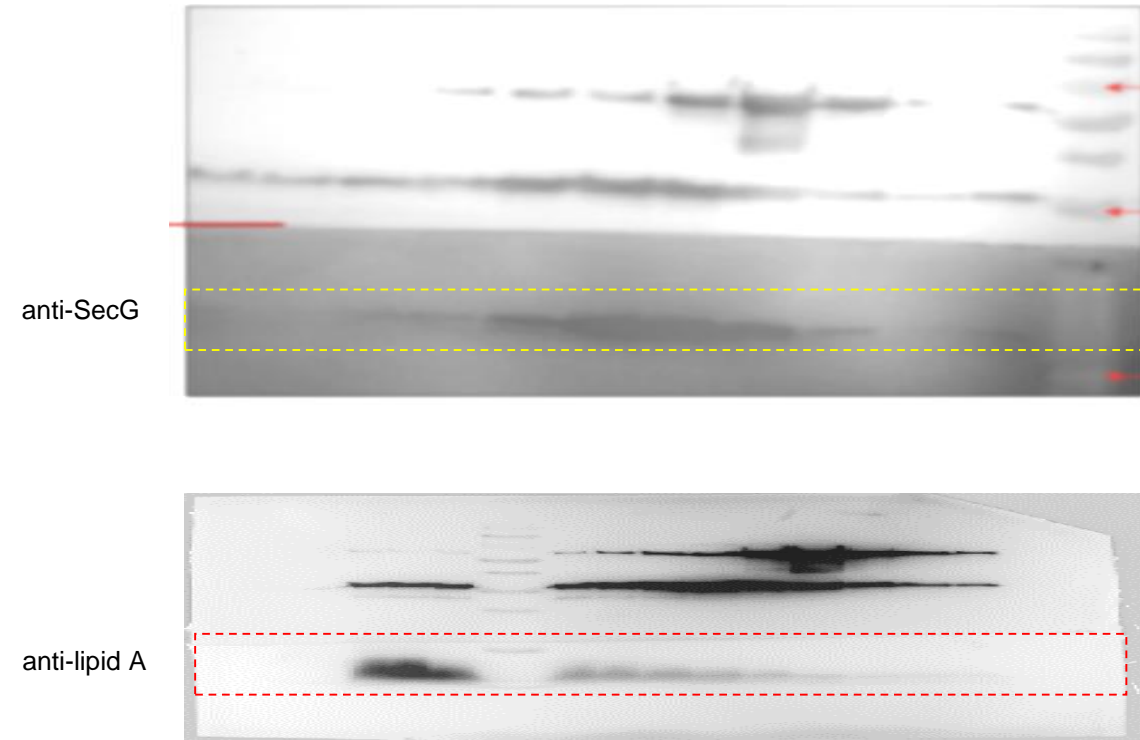

Figure 1c

Supplement: Figure 1—source data 3. [file elife-72824-fig1-data3.pdf]

original

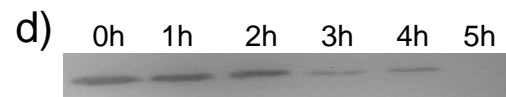

uncropped

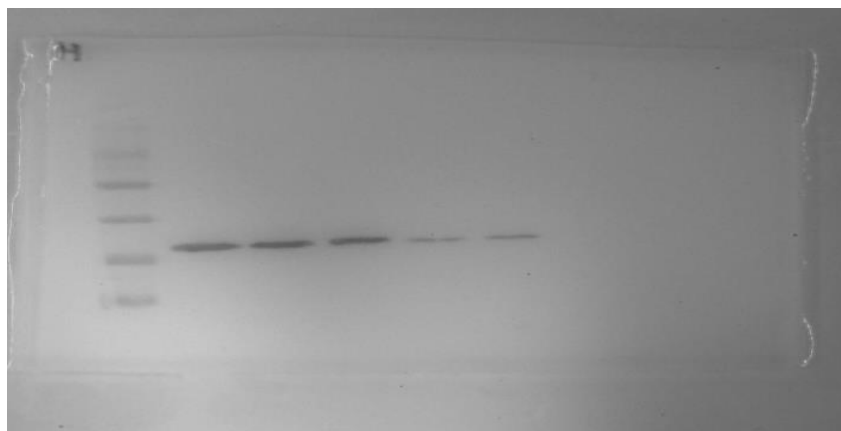

Figure 1d

Supplement: Figure 1—source data 4. [file elife-72824-fig1-data4.pdf]

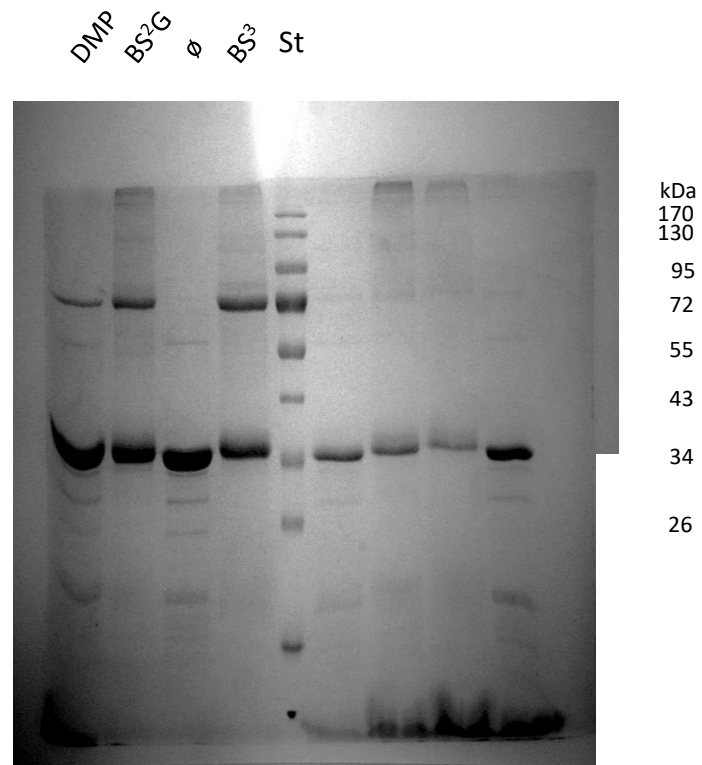

Figure 5b

Supplement: Figure 5—source data 2. [file elife-72824-fig5-data2.pdf]

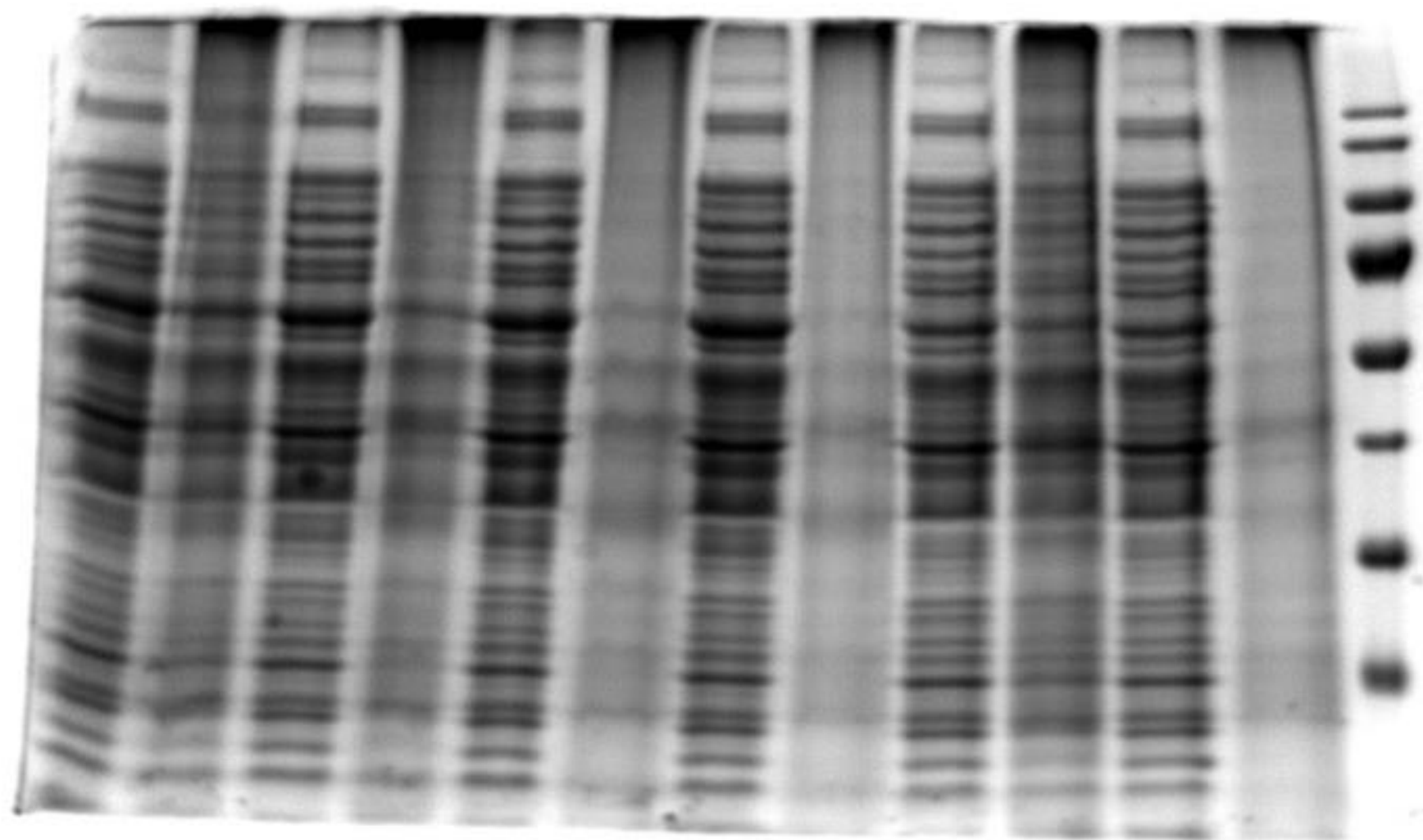

Supplement: Figure 5—figure supplement 1—source data 2. [file elife-72824-fig5-figsupp1-data2.pdf]

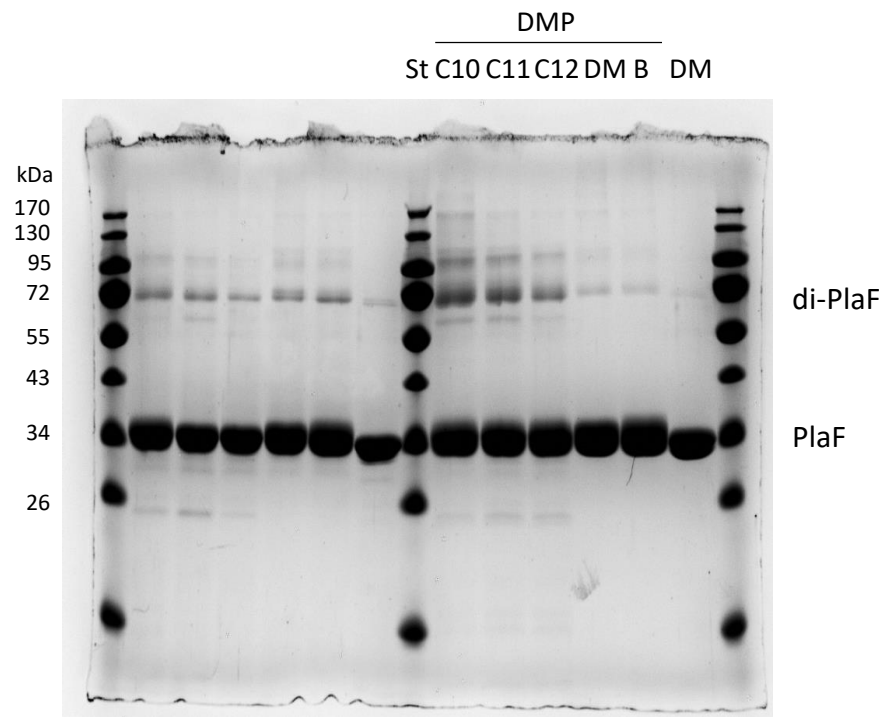

Figure 6c

Supplement: Figure 6—source data 3. [file elife-72824-fig6-data3.pdf]

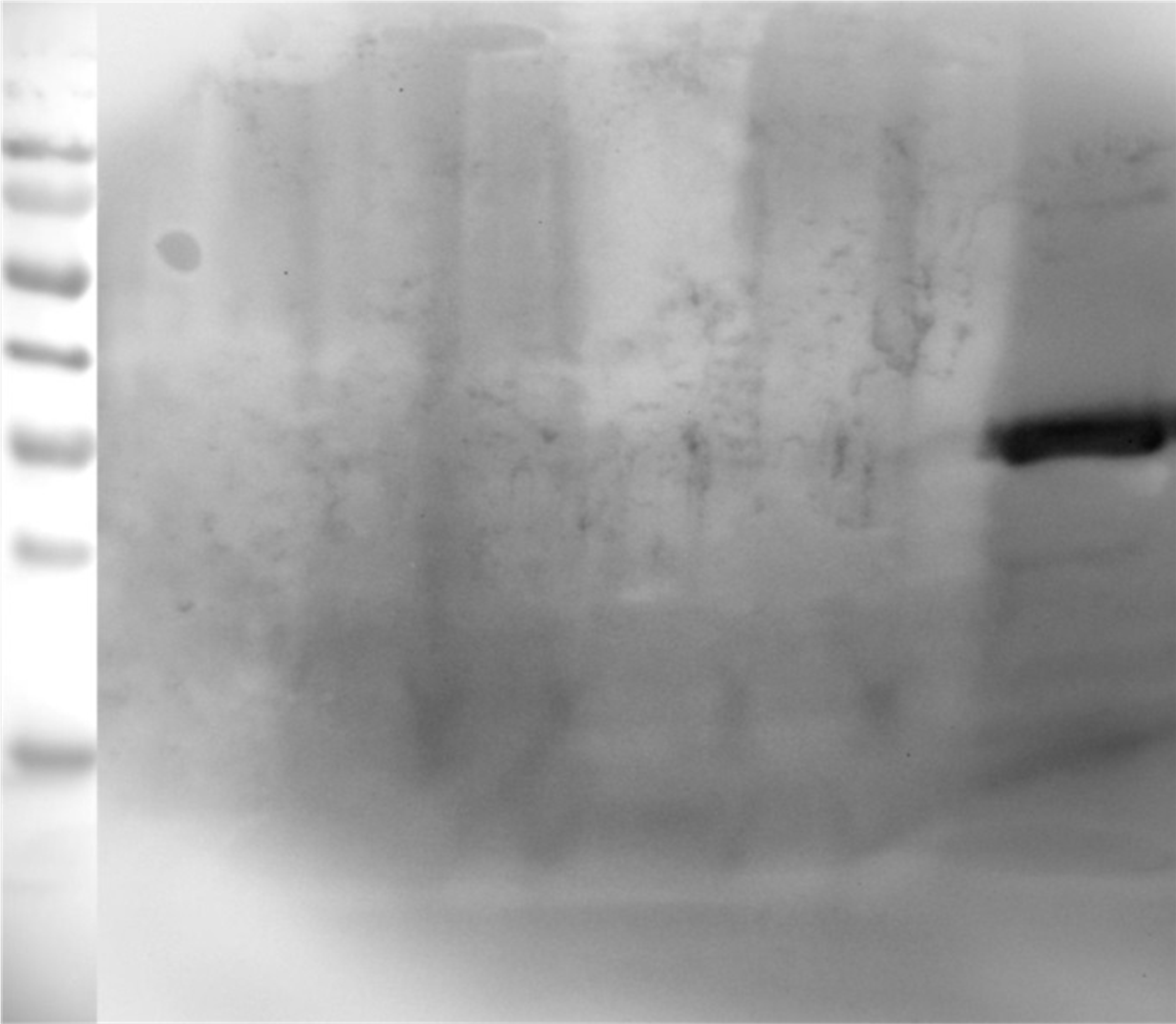

Supplement: Figure 7—figure supplement 2—source data 1. [file elife-72824-fig7-figsupp2-data1.tif]

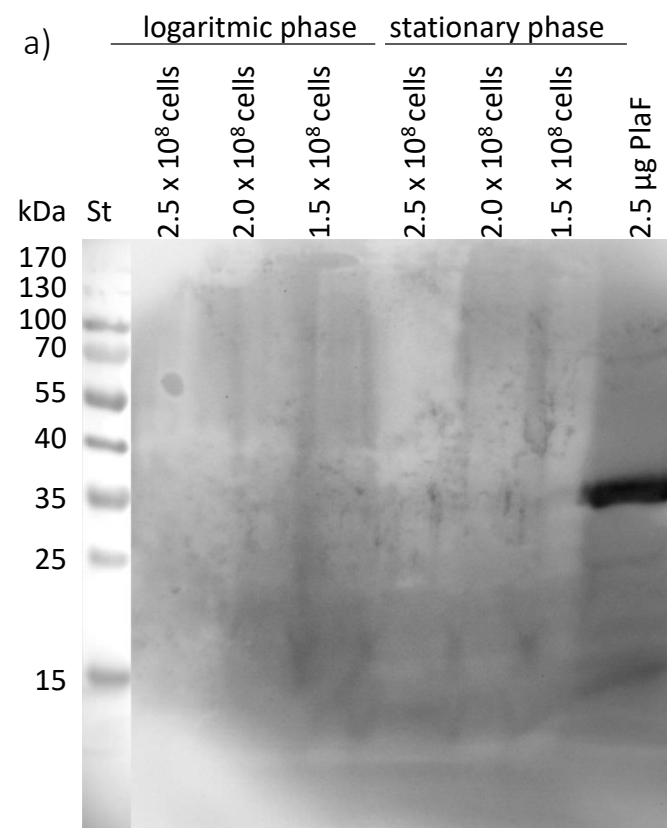

Supplement: Figure 7—figure supplement 2—source data 2. [file elife-72824-fig7-figsupp2-data2.pdf]

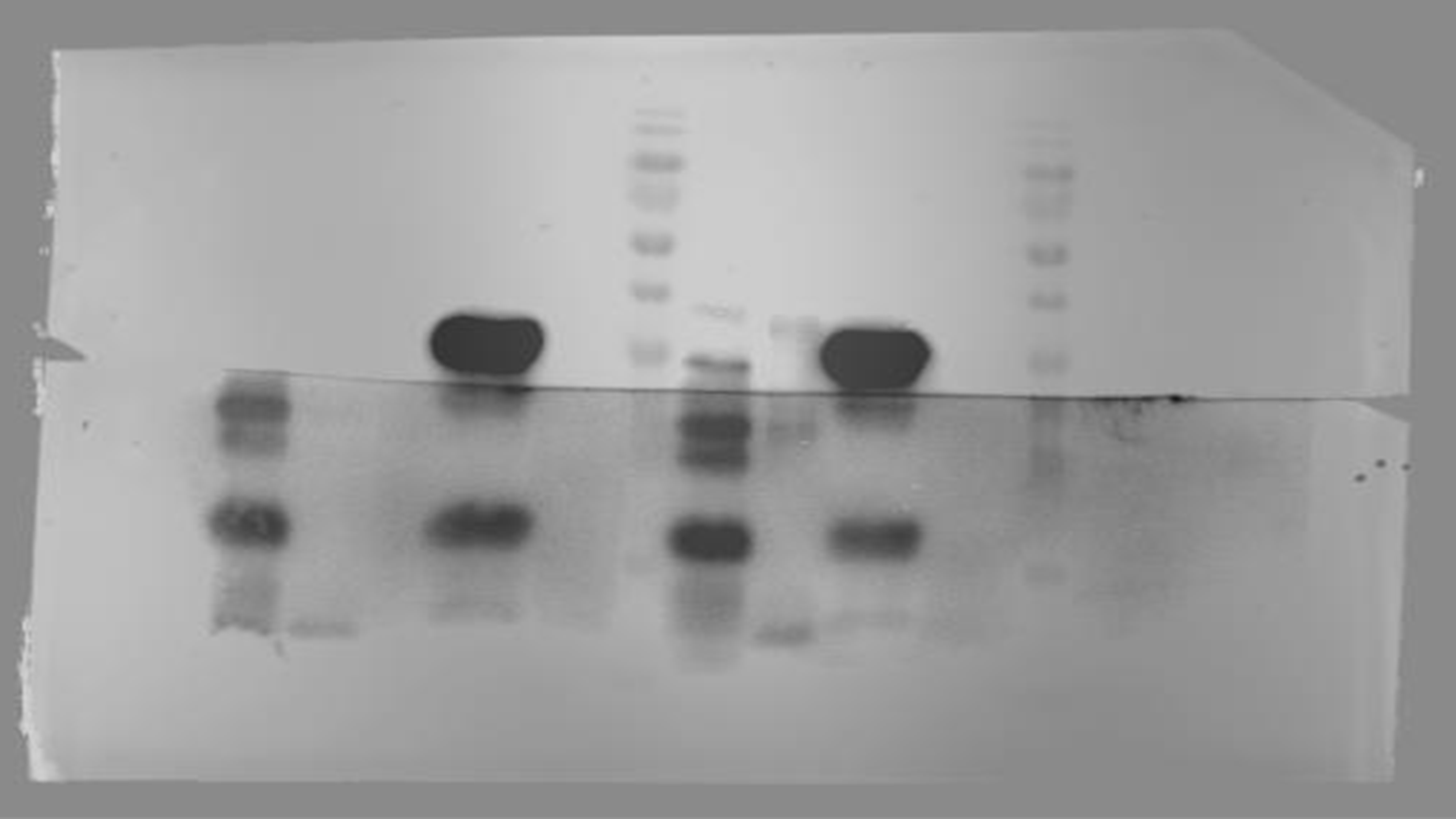

Supplement: Source data 1. [file elife-72824-data1.zip › Figure 1-source data 1a.tif]

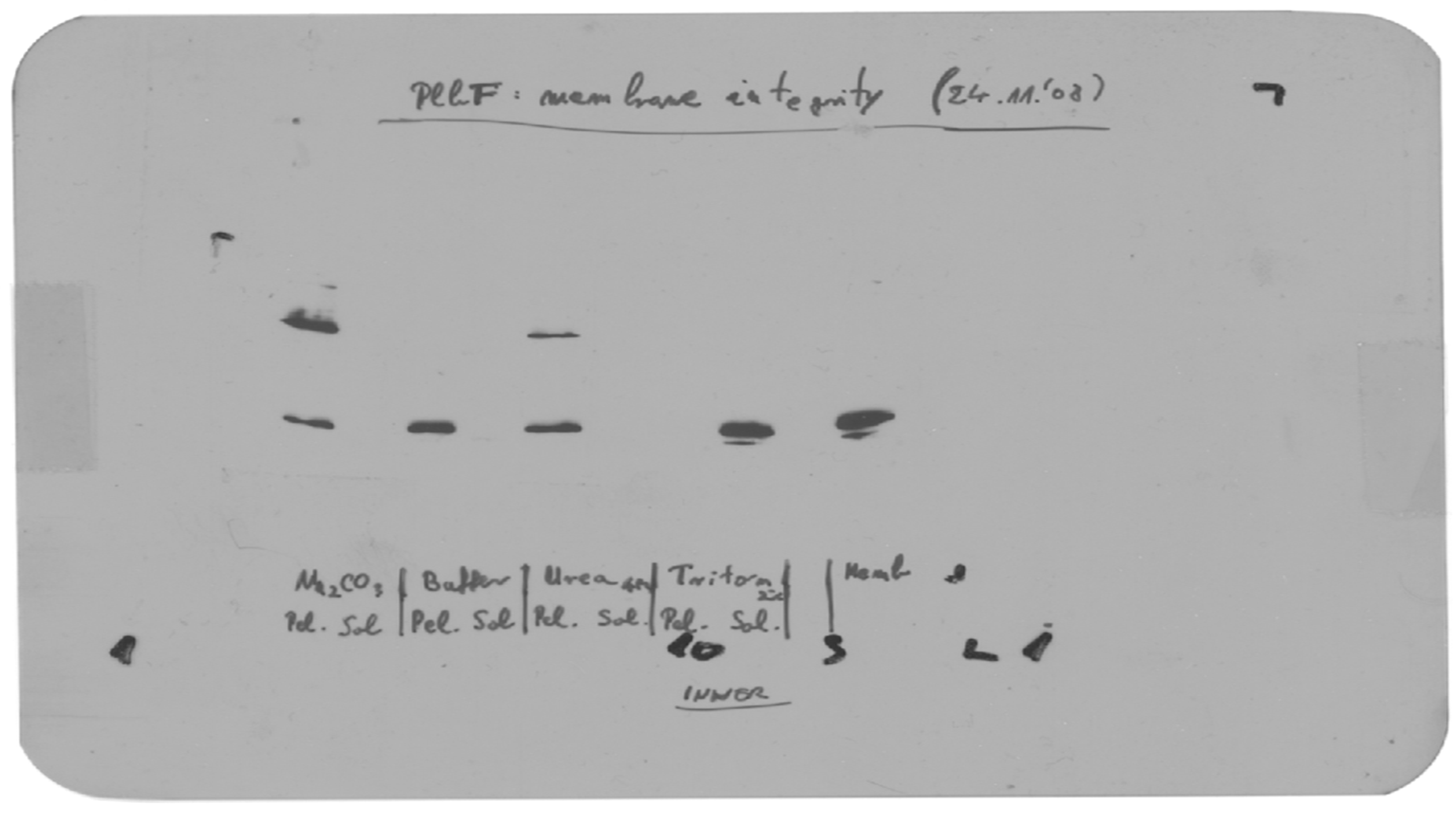

Supplement: Source data 1. [file elife-72824-data1.zip › Figure 1-source data 2a.tif]

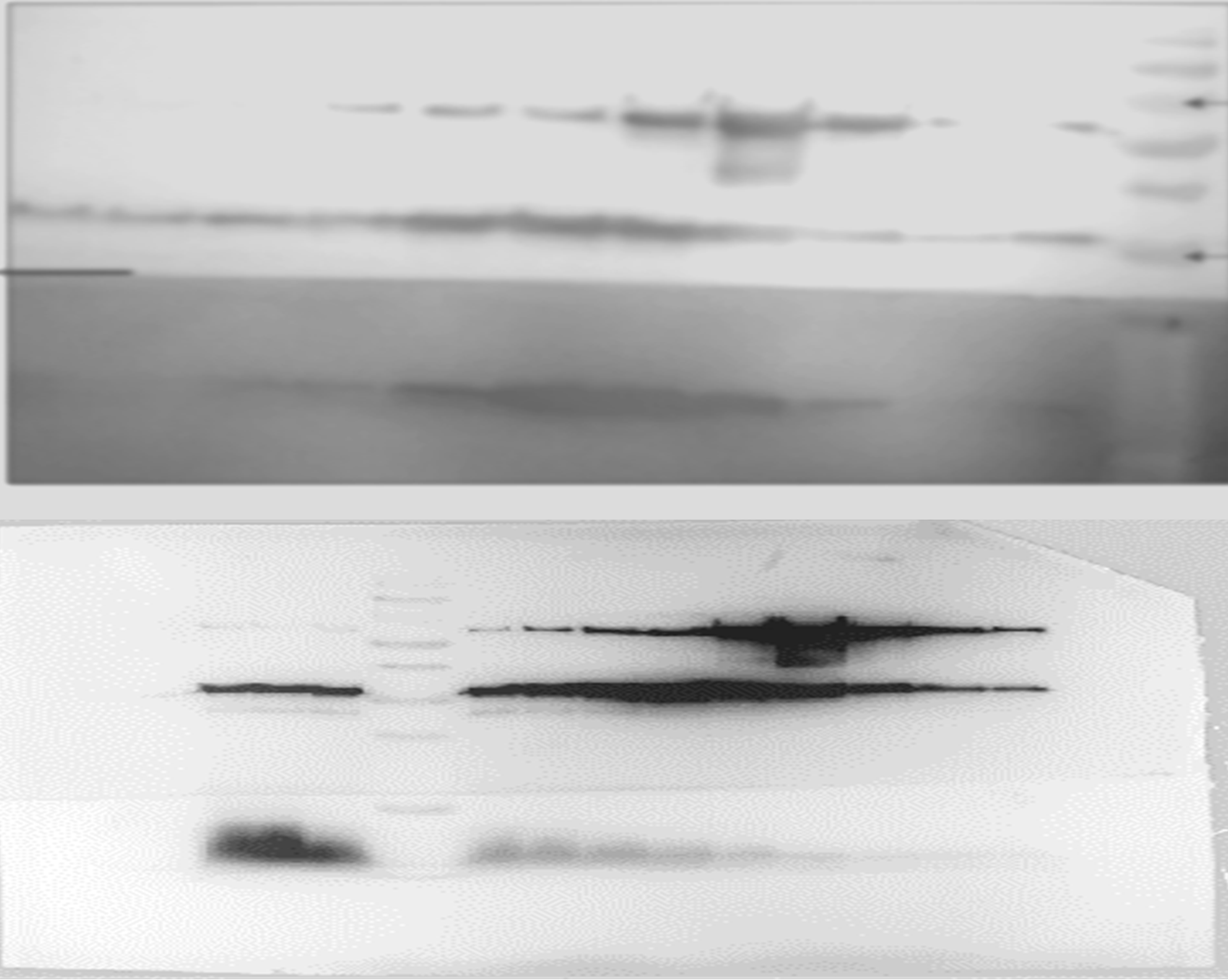

Supplement: Source data 1. [file elife-72824-data1.zip › Figure 1-source data 3a.tif]

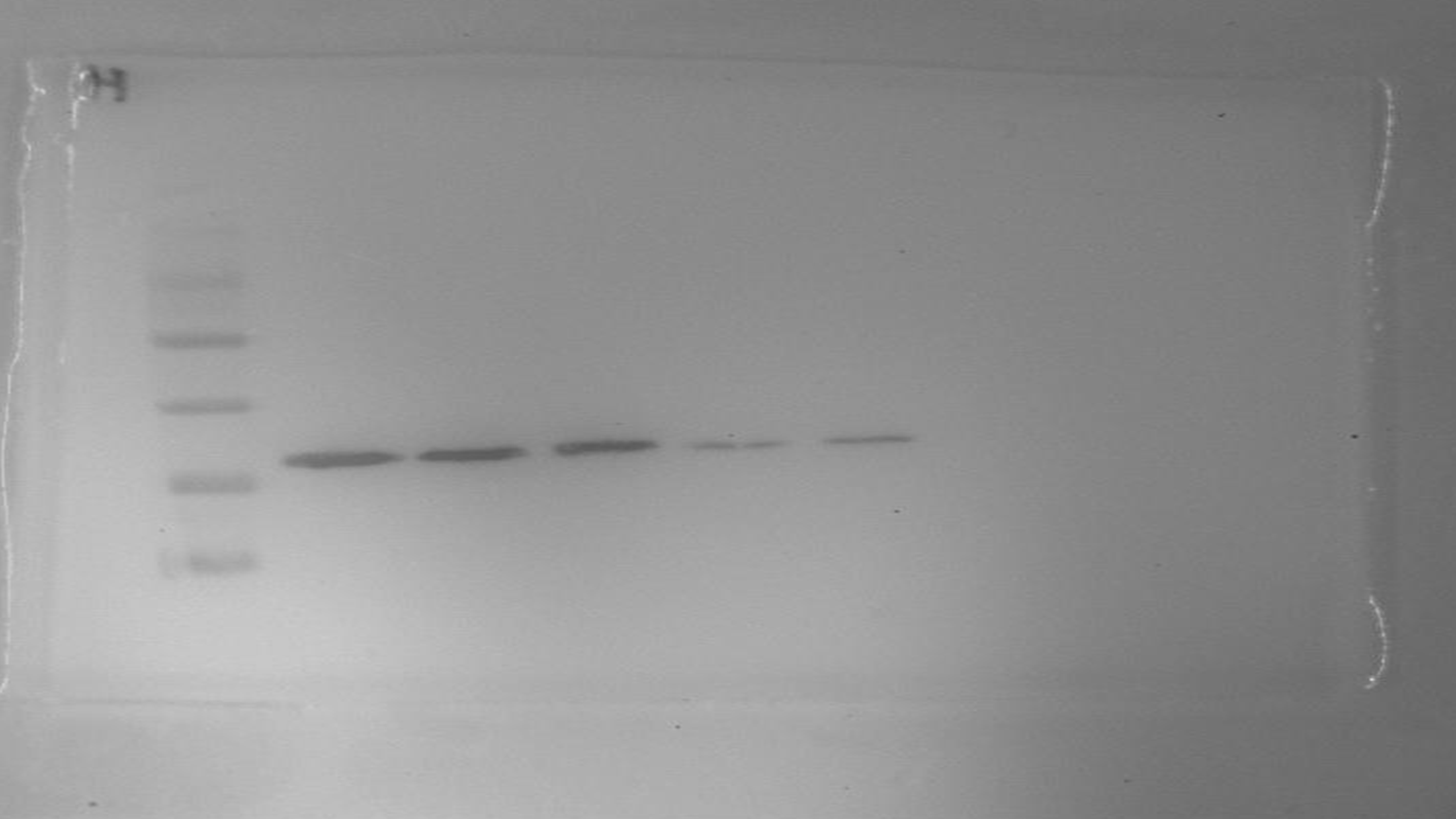

Supplement: Source data 1. [file elife-72824-data1.zip › Figure 1-source data 4a.tif]

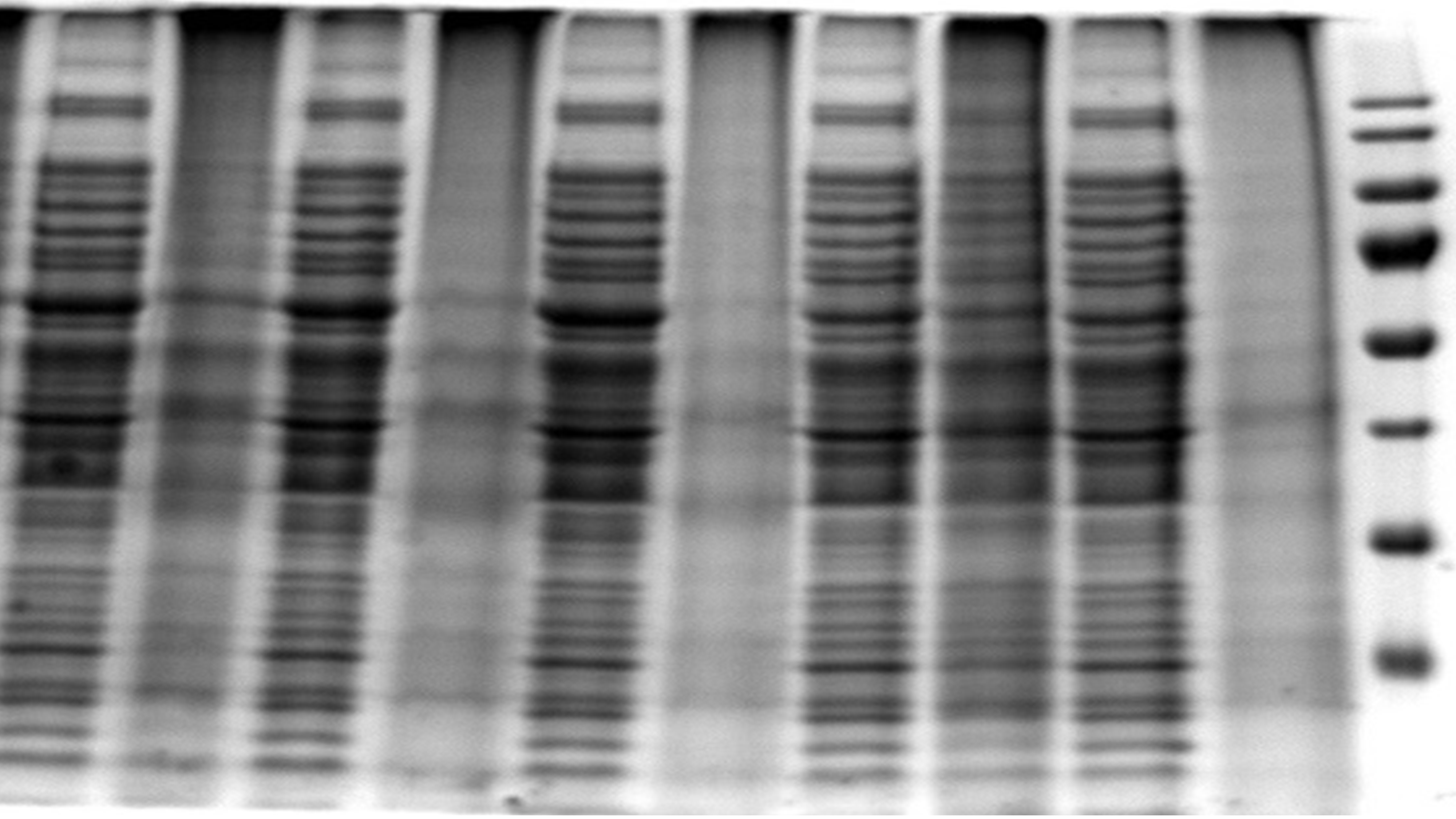

Supplement: Source data 1. [file elife-72824-data1.zip › Figure 5-figure supplement 1-source data 1.tif]

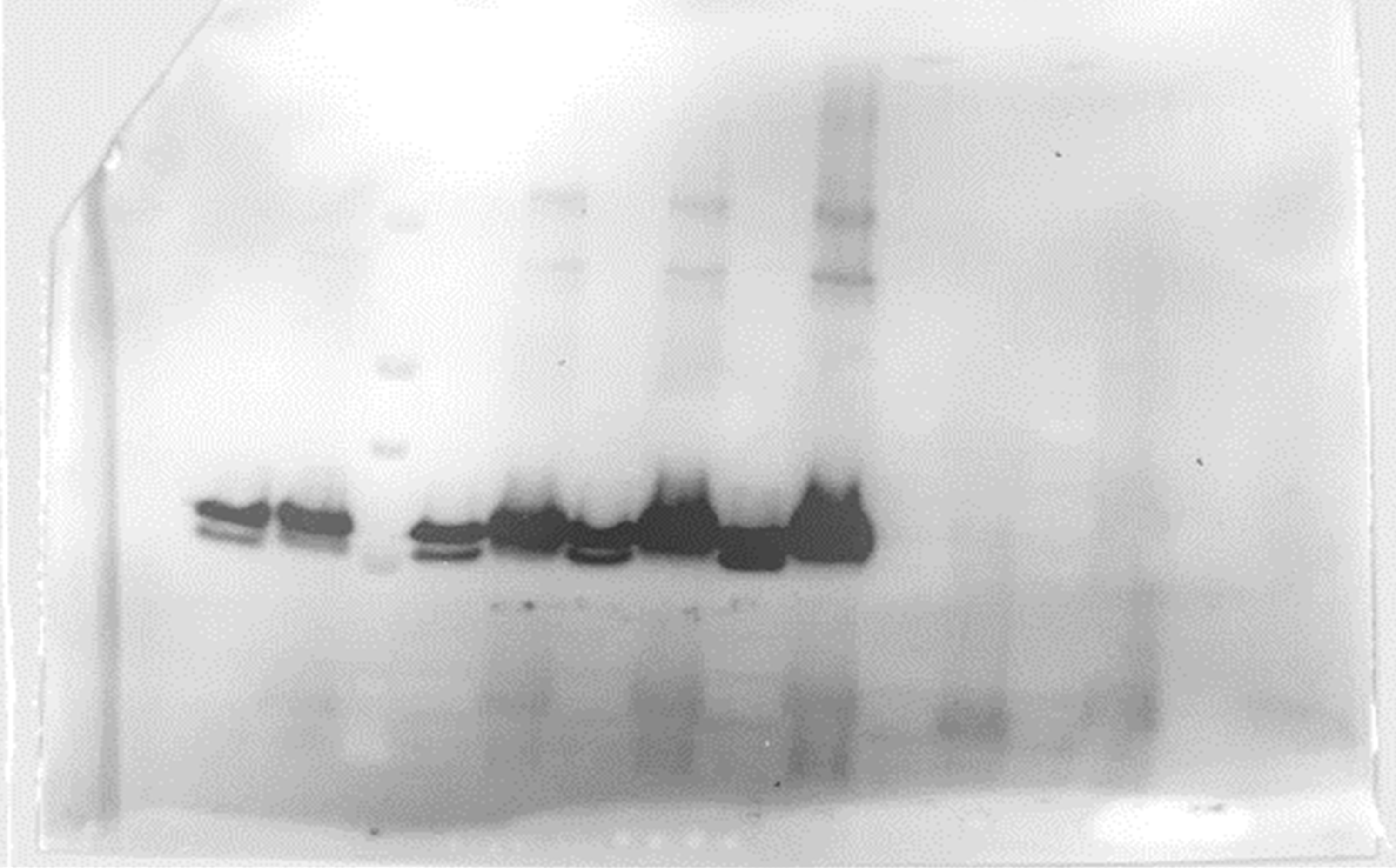

Supplement: Source data 1. [file elife-72824-data1.zip › Figure 5-source data 1a.tif]

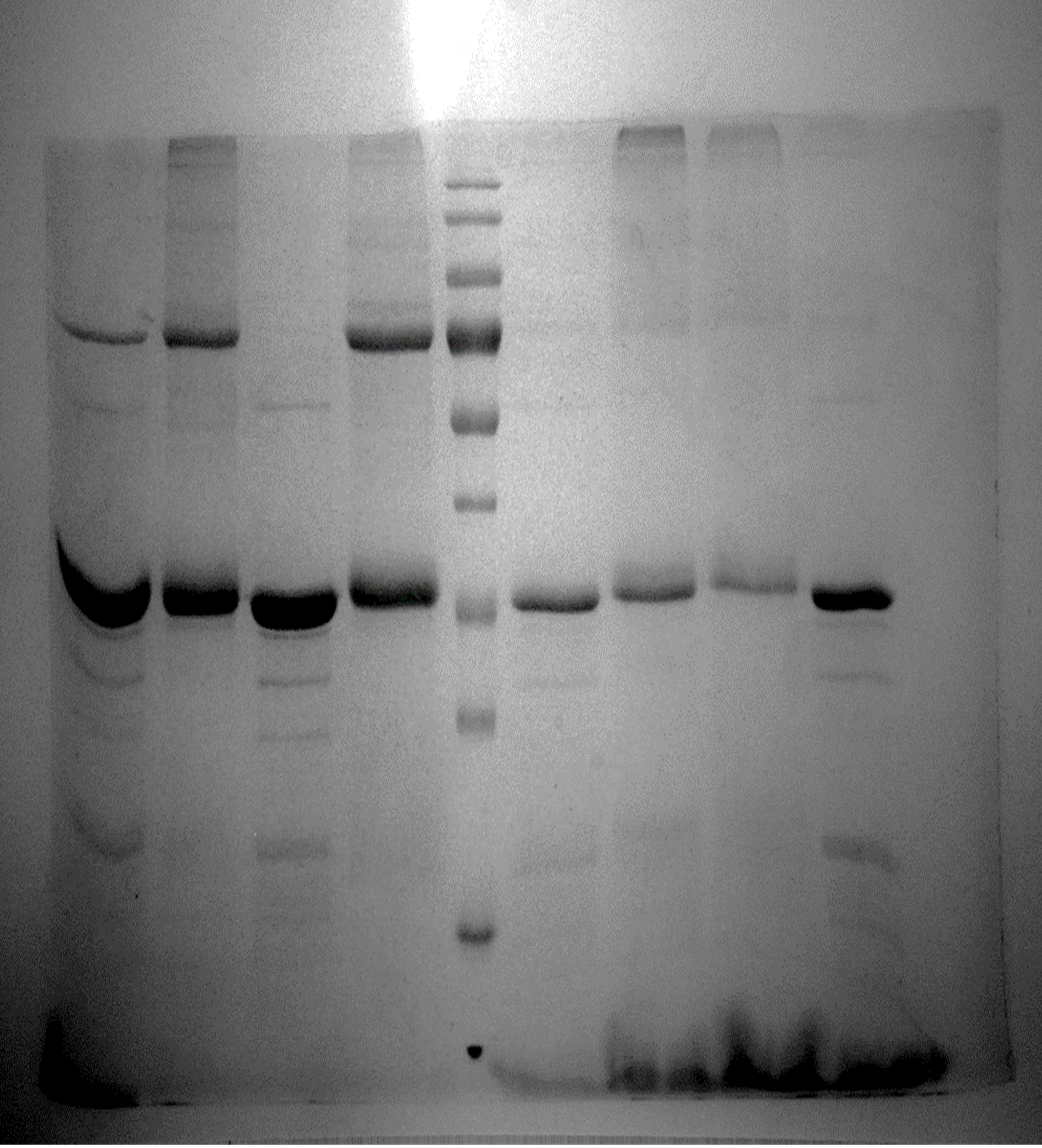

Supplement: Source data 1. [file elife-72824-data1.zip › Figure 5-source data 2a.tif]

## Slide 1
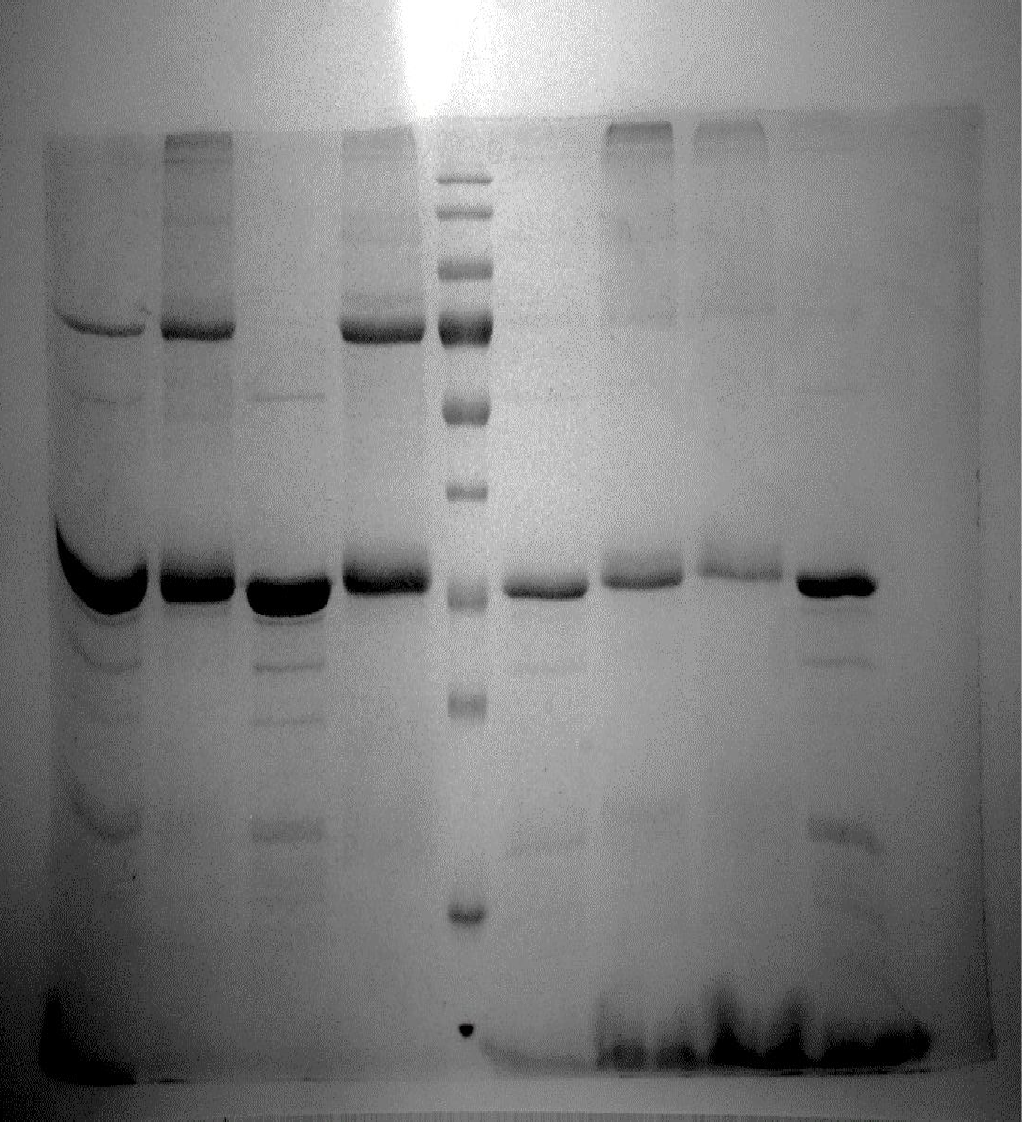

Supplement: Source data 1. [file elife-72824-data1.zip › Figure 5b.pptx]

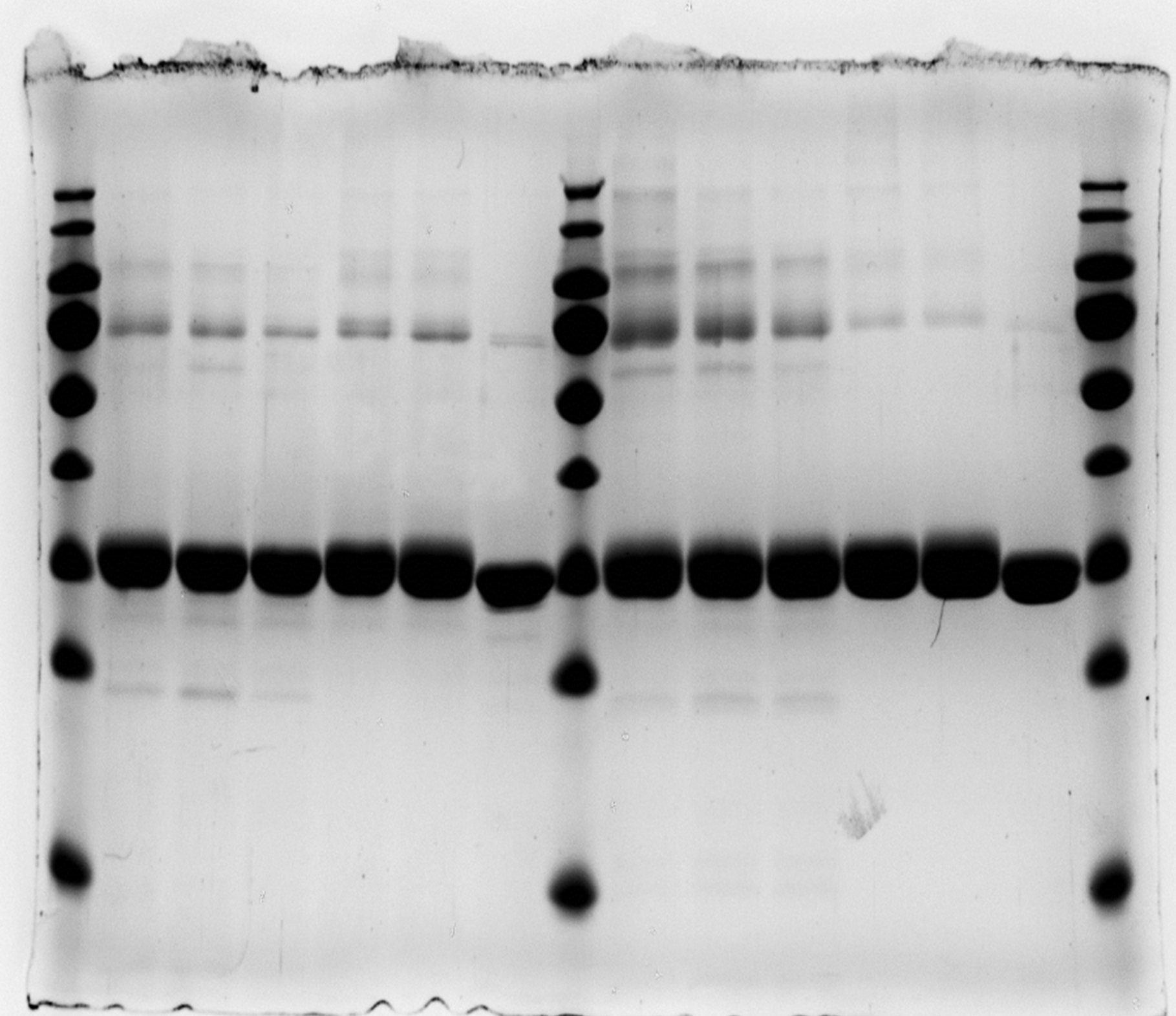

Supplement: Source data 1. [file elife-72824-data1.zip › Figure 6-source data 2a.tif]
