# Supplementary material for: Structural, mechanistic, and physiological insights into phospholipase A-mediated membrane phospholipid degradation in Pseudomonas aeruginosa
Source: eLife. 2022 May 10;11:e72824. doi: 10.7554/eLife.72824 (PMC9132575; doi:10.7554/eLife.72824)
Supplement: Supplementary file 2. [file elife-72824-supp2.docx]

**Supplementary File 2:** Phospholipid species significantly differentially abundant in *P. aeruginosa* wild-type, ∆*plaF,* and ∆*plaF*::*plaF*.

| GPL^#^ | *P. aeruginosa* PAO1 nmol/mg(GPL)/OD_580nm_ ± SD | | | *P. aeruginosa* Δ*plaF*  nmol/mg(GPL)/OD_580nm_ ± SD T-test^*^ | | | | *P. aeruginosa* Δ*plaF*::*plaF*  nmol/mg(GPL)/OD_580nm_ ± SD T-test^*^ | | | |
| --- | --- | --- | --- | --- | --- | --- | --- | --- | --- | --- | --- |
| PI 26:0 | 0.0018 | ± | 0.0007 | 0.0039 | ± | 0.0013 | 0.032 | 0.0033 | ± | 0.0009 | 0.076 |
| PE 31:2 | 0.0007 | ± | 0.0004 | 0.0019 | ± | 0.0007 | 0.040 | 0.0014 | ± | 0.0006 | 0.189 |
| PG 36:0 | 0.0006 | ± | 0.0005 | 0.0014 | ± | 0.0003 | 0.042 | 0.0007 | ± | 0.0003 | 0.699 |
| PE 22:1 | 0.0001 | ± | 0.0001 | 0.0006 | ± | 0.0002 | 0.019 | 0.0004 | ± | 0.0001 | 0.007 |
| PG 24:3 | 0.0002 | ± | 0.0001 | 0.0005 | ± | 0.0001 | 0.033 | 0.0002 | ± | 0.0001 | 0.907 |
| PC 39:0 | 0.0001 | ± | 0.0000 | 0.0002 | ± | 0.0000 | 0.040 | 0.0002 | ± | 0.0001 | 0.181 |
| PC 36:1 | 0.0005 | ± | 0.0001 | 0.0003 | ± | 0.0001 | 0.021 | 0.0004 | ± | 0.0001 | 0.175 |
| PG 23:2 | 0.0004 | ± | 0.0001 | 0.0001 | ± | 0.0001 | 0.009 | 0.0003 | ± | 0.0001 | 0.318 |
| PE 26:3 | 0.0007 | ± | 0.0000 | 0.0003 | ± | 0.0001 | 0.004 | 0.0005 | ± | 0.0002 | 0.255 |
| PC 33:1 | 0.0009 | ± | 0.0002 | 0.0004 | ± | 0.0001 | 0.004 | 0.0005 | ± | 0.0002 | 0.021 |
| PE 37:4 | 0.0024 | ± | 0.0004 | 0.0014 | ± | 0.0004 | 0.016 | 0.0013 | ± | 0.0003 | 0.015 |
| PC 32:0 | 0.0022 | ± | 0.0004 | 0.0010 | ± | 0.0004 | 0.004 | 0.0013 | ± | 0.0004 | 0.042 |
| PC 35:2 | 0.0021 | ± | 0.0003 | 0.0007 | ± | 0.0004 | 0.002 | 0.0011 | ± | 0.0005 | 0.047 |
| PC 32:1 | 0.0022 | ± | 0.0009 | 0.0007 | ± | 0.0002 | 0.033 | 0.0012 | ± | 0.0004 | 0.101 |
| PG 32:0 | 0.0033 | ± | 0.0009 | 0.0016 | ± | 0.0001 | 0.031 | 0.0032 | ± | 0.0007 | 0.806 |
| PC 35:1 | 0.0071 | ± | 0.0010 | 0.0035 | ± | 0.0018 | 0.017 | 0.0045 | ± | 0.0022 | 0.161 |
| PE 33:0 | 0.0092 | ± | 0.0009 | 0.0052 | ± | 0.0016 | 0.009 | 0.0061 | ± | 0.0018 | 0.082 |
| PC 34:1 | 0.0281 | ± | 0.0077 | 0.0117 | ± | 0.0045 | 0.015 | 0.0140 | ± | 0.0034 | 0.028 |
| PE 33:1 | 0.0546 | ± | 0.0117 | 0.0283 | ± | 0.0116 | 0.019 | 0.0324 | ± | 0.0065 | 0.026 |
| PG 35:1 | 0.0570 | ± | 0.0092 | 0.0269 | ± | 0.0144 | 0.016 | 0.0319 | ± | 0.0086 | 0.016 |
| PE 35:1 | 0.4968 | ± | 0.0869 | 0.2503 | ± | 0.1635 | 0.049 | 0.2656 | ± | 0.0804 | 0.017 |
| **Total^€^** | **0.6711** | **±** | **0.1223** | **0.3409** | **±** | **0.2021** | **-** | **0.3705** | **±** | **0.1080** | **-** |

^#^ Phospholipid nomenclature XX:Y; XX, the sum of carbon atoms in fatty acids bound to phospholipid; Y, the number of double bonds in fatty acids bound to phospholipid.

* Significance compared to the *P. aeruginosa* PAO1.

^€^ Total amount of significantly changed GPLs.
