# Supplementary material for: Structural, mechanistic, and physiological insights into phospholipase A-mediated membrane phospholipid degradation in Pseudomonas aeruginosa
Source: eLife. 2022 May 10;11:e72824. doi: 10.7554/eLife.72824 (PMC9132575; doi:10.7554/eLife.72824)
Supplement: Supplementary file 3. [file elife-72824-supp3.docx]

**Supplementary File 3:** Properties of the cultures used for lipid extraction.

|  | Biological replicate | *P. aeruginosa* PA01 | *P. aeruginosa* ∆*plaF* | *P. aeruginosa* ∆*plaF*::*plaF* |
| --- | --- | --- | --- | --- |
| Optical density [OD_580nm_] | 1 | 5.93 | 5.99 | - |
|  | 2 | 7.11 | 4.24 | 5.07 |
|  | 3 | 6.54 | 6.03 | 5.02 |
|  | 4 | 6.13 | 4.96 | 4.75 |
| GPL [mg]* | 1 | 54.7 | 64.1 | - |
|  | 2 | 65.5 | 63.4 | 64.6 |
|  | 3 | 65.1 | 63.8 | 64.7 |
|  | 4 | 64.9 | 63.9 | 64.5 |
| T-test** |  | - | 0.67 | 0.49 |

* Isolated from 15 ml culture.

** Significance compared to the *P. aeruginosa* PAO1.
