# Supplementary material for: Structural, mechanistic, and physiological insights into phospholipase A-mediated membrane phospholipid degradation in Pseudomonas aeruginosa
Source: eLife. 2022 May 10;11:e72824. doi: 10.7554/eLife.72824 (PMC9132575; doi:10.7554/eLife.72824)
Supplement: Supplementary file 5. [file elife-72824-supp5.docx]

**Supplementary File 5:** List of interactions involving the ligand molecules.

| Ligand | Interacting residue in PlaFA | | | | Ligand | Interacting residue in PlaFB | | | |
| --- | --- | --- | --- | --- | --- | --- | --- | --- | --- |
| Atom | Distance / Å | Atom | Residue | # | Atom | Distance / Å | Atom | Residue | # |
| MYR | | | | | 11A | | | | |
| O1 | 3.28 | OD1 | ASN | 136 | C3 | 3.62 | OD1 | ASN | 77 |
| O1 | 3.47 | O | HOH | 113 | C3 | 3.34 | O | HOH | 90 |
| O1 | 3.43 | OGB | SER | 137 | C4 | 3.51 | C8' | BOG | 502 |
| C1 | 3.64 | O | PHE | 71 | C4 | 3.65 | O | HOH | 90 |
| C3 | 3.20 | OD1 | ASN | 77 | C5 | 3.57 | C5' | BOG | 502 |
| C3 | 3.89 | CB | ALA | 73 | C5 | 3.74 | C8' | BOG | 502 |
| C3 | 3.67 | CG1 | VAL | 287 | C6 | 3.04 | O | HOH | 90 |
| C4 | 3.47 | OD1 | ASN | 77 | C7 | 3.54 | O | HOH | 90 |
| C7 | 3.66 | ND2 | ASN | 77 | C10 | 3.67 | CA | ARG | 31 |
| C8 | 3.83 | C7' | BOG | 502 | C10 | 3.76 | CG | ARG | 31 |
| C10 | 3.11 | O | HOH | 166 | C10 | 3.64 | CD1 | LEU | 79 |
| O2 | 3.43 | O | PHE | 71 | O1 | 3.12 | ND2 | ASN | 77 |
| OG | | | | | O1 | 2.99 | O2 | IPA | 504 |
| O1 | 3.48 | NH2 | ARG | 80 | C | 3.45 | O2 | IPA | 504 |
| C5' | 3.73 | CD1 | LEU | 206 | O | 3.39 | O2 | IPA | 504 |
| C8' | 3.85 | CE2 | PHE | 200 | C1 | 3.56 | OD1 | ASN | 77 |
| O6 | 2.58 | OE2 | GLU | 34 | C2 | 3.62 | OD1 | ASN | 77 |
| O6 | 3.56 | CD | GLU | 34 | IPA503 | | | | |
| C3' | 3.73 | NH2 | ARG | 80 | C1 | 3.16 | OD1 | ASN | 136 |
| C3' | 3.69 | O | HOH | 28 | C1 | 3.49 | O | HOH | 22 |
| C1' | 3.63 | CG1 | VAL | 30 | C1 | 3.25 | OGB | SER | 137 |
| C7' | 3.72 | CE2 | PHE | 200 | C2 | 3.28 | O | PHE | 71 |
| C7' | 3.74 | CZ | PHE | 200 | O2 | 3.45 | C | 11A | 501 |
| C7' | 3.83 | C8 | MYR | 500 | O2 | 3.39 | O | 11A | 501 |
| C8' | 3.88 | CD | GLN | 203 | O2 | 2.99 | O1 | 11A | 501 |
| C8' | 3.56 | CG | GLN | 203 | C3 | 3.75 | CD2 | HIS | 286 |
| C8' | 3.62 | NE2 | GLN | 203 | C3 | 3.69 | NE2 | HIS | 286 |
| C8' | 3.57 | O | PRO | 204 | C3 | 3.32 | O | HOH | 171 |
| O4 | 3.42 | O | HOH | 105 | OG | | | | |
| C2 | 3.67 | O | PRO | 205 | O1 | 2.99 | NH2 | ARG | 80 |
| O2 | 2.96 | O | PRO | 205 | C5' | 3.82 | CD1 | LEU | 206 |
| O2 | 3.23 | CD | PRO | 207 | C8' | 3.87 | CE2 | PHE | 200 |
| O2 | 3.51 | C | PRO | 205 | O6 | 2.52 | OE2 | GLU | 34 |
| O2 | 3.70 | CG | PRO | 207 | O6 | 3.42 | CD | GLU | 34 |
| C6 | 3.67 | O | HOH | 49 | C3' | 3.78 | C3 | IPA | 503 |
| O6 | 3.42 | CG1 | VAL | 33 | C2' | 3.73 | CD2 | LEU | 210 |
|  |  |  |  |  | C4' | 3.62 | NH1 | ARG | 80 |
|  |  |  |  |  | O1 | 3.36 | O | HOH | 147 |
|  |  |  |  |  | C5' | 3.57 | C5 | 11A | 501 |
|  |  |  |  |  | C6' | 3.81 | CB | PRO | 204 |
|  | van-der-waals | |  |  | C1 | 3.70 | NH2 | ARG | 80 |
|  | electrostatic | |  |  | C8' | 3.51 | C4 | 11A | 501 |
|  | hydrogen | |  |  | C8' | 3.74 | C5 | 11A | 501 |
|  | ionic | |  |  | O5 | 3.14 | OE2 | GLU | 34 |
|  |  |  |  |  | C5 | 3.90 | CG1 | VAL | 30 |
|  |  |  |  |  | C6 | 3.43 | OE2 | GLU | 34 |
|  |  |  |  |  | O6 | 3.68 | CG | GLU | 34 |
