# Supplementary material for: Structural, mechanistic, and physiological insights into phospholipase A-mediated membrane phospholipid degradation in Pseudomonas aeruginosa
Source: eLife. 2022 May 10;11:e72824. doi: 10.7554/eLife.72824 (PMC9132575; doi:10.7554/eLife.72824)
Supplement: Supplementary file 6. [file elife-72824-supp6.docx]

**Supplementary File 6:** List of interactions involving the dimer interface.

| Source atoms | Target atoms | Distance (Å) |
| --- | --- | --- |
| Leu 5A CG  Leu 5A CD1  Leu 8A CG  Leu 8A CD2  Val 9A CA  Val 9A C  Val 9A O  Val 9A CG1  Val 9A CG2  Leu 12A C  Leu 12A CB  Leu 12A CD1  Ala 13A N  Ala 13A CA  Ala 13A C  Ala 13A O  Ala 13A CB  Val 14A N  Val 14A CA  Val 14A C  Val 14A O  Gly 17A N  Gly 17A CA  Gly 17A C  Gly 17A O  Val 18A N  Val 18A CG2  Phe 21A CE1  Phe 21A CZ  Phe 21A CE2  Phe 21A CD2  Val 22A CG2  Thr 25A CB  Thr 25A OG1  Thr 25A CG2  Ser 29A O  Ser 29A CA  Ser 29A CB  Ser 29A OG  Ser 29A CA  Ser 29A CB  Ser 29A OG  Thr 32A CB  Thr 32A CG2  Val 33A CB  Val 33A CG1  Val 33A CG2  Val 33A O  Leu 37A CG  Leu 37A CD1  Leu 37A CD2 | Val 9B CG2  Val 9B CG2  Val 9B CG1  Val 9B CG1  Val 9B CG2  Ala 13B CB  Ala 13B CB  Ala 13B CA  Ala 13B CB  Leu 12B C  Leu 12B O  Ala 13B N  Ala 13B CA  Ala 13B CB  Leu 12B CB  Ala 16B CB  Val 9B O  Leu 12B CB  Val 9B CA  Val 9B CG1  Ala 13B CB  Ala 13B CB  Ala 13B CB  Leu 10B CD2  Val 9B CG1  Ala 13B CB  Ala 13B O  Gly 17B CA  Ala 13B C  Ala 13B O  Phe 21B CE2  Phe 21B CD2  Phe 21B CE2  Phe 21B CZ  Gly 17B CA  Ala 16B C  Gly 17B N  Gly 17B CA  Ala 13B C  Ala 13B O  Ala 16B CB  Phe 21B CE2  Phe 21B CE2  Phe 21B CZ  Phe 21B CE2  Phe 21B CZ  Phe 21B CE2  Phe 21B CZ  Phe 21B CD2  Phe 21B CE2  Phe 21B CG  Phe 21B CD2  Phe 21B CD1  Phe 21B CE2  Phe 21B CZ  Phe 21B CE1  Phe 21B CG  Phe 21B CD2  Phe 21B CD1  Phe 21B CE2  Phe 21B CZ  Phe 21B CE1  Phe 21B CD1  Phe 21B CE1  Phe 21B CE2  Phe 21B CZ  Phe 21B CE1  Phe 21B CZ  Phe 21B CE1  Val 22B CG2  Val 22B CA  Val 22B CB  Thr 25B OG1  Val 22B CG2  Val 22B CA  Val 22B CB  Phe 21B C  Phe 21B O  Val 22B N  Thr 25B CB  Thr 25B OG1  Thr 25B CG2  Val 22B CG2  Phe 21B C  Phe 21B O  Val 22B N  Thr 25B CB  Thr 25B OG1  Thr 25B CG2  Val 22B CG2  Phe 21B CB  Thr 25B CG2  Phe 21B CD1  Phe 21B CE1  Thr 25B CB  Thr 25B OG1  Thr 25B CB  Thr 25B OG1  Thr 25B CG2  Thr 25B CB  Thr 25B OG1  Val 33B CG2  Val 33B CG2  Val 33B CG2  Thr 32B CG2  Ser 29B CB  Thr 32B CB  Ser 29B CA  Ser 29B CB  Ser 29B OG  Ser 29B C  Ser 29B O  Thr 32B CB  Val 33B CG2  Val 33B CG2  Thr 32B CG2  Ser 29B CB  Thr 32B CB  Thr 32B CG2  Thr 32B CB  Val 33B CG2  Val 33B CG1  Val 33B CG2  Gly 36B CA  Gly 36B O  Leu 37B N  Gly 36B CA  Gly 36B C  Val 33B C  Val 33B O  Leu 37B N  Gly 36B CA  Gly 36B C  Thr 32B C  Val 33B N  Val 33B CA  Val 33B CB  Val 33B CG1  Val 33B CG2  Thr 32B O  Leu 37B CD2  Leu 37B CD2  Gly 36B O  Arg 83B NH2  Leu 37B CD2  Leu 37B O  Leu 37B CA  Leu 37B C | 3.95  4.49  3.87  3.89  4.43  4.42  4.45  3.97  3.66  4.09  4.10  3.94  3.80  4.38  4.41  4.17  3.88  4.33  4.45  4.13  4.41  3.76  4.48  3.62  3.84  4.17  4.44 *  4.43  4.46  3.72  4.14  3.84  3.42  4.45  4.24  4.38  3.86  4.02  4.38  3.51  4.17  4.27  3.66  4.06  4.13  4.29  3.78  3.63  4.27  4.07  3.77  3.54  3.97  3.52  3.75  3.96  4.43  4.45  4.13  4.19  3.89  3.86  4.33  4.25  4.29  3.78  4.00  4.15  4.47  4.38  4.23  4.42  3.95  3.69  3.68  4.28  4.02  3.93  3.93  4.15  3.03  4.24  3.75  4.23  4.28  4.36  4.44  3.69  4.08  4.48  3.90  3.87  3.68  3.74  4.46  4.31  3.88  4.16 *  4.43  4.33  3.78  3.97  4.26  4.27  3.75  4.43  4.13  3.64  3.15  3.55 *  4.29  4.09 *  4.45  4.26  4.25  3.77  4.40  4.13  3.52  4.13  4.16  4.05  3.93  4.45  3.58  3.90  3.66  3.46  4.18  3.81  4.35  3.95  4.32  4.47  4.37  3.58  4.29  4.38  4.27  3.90  4.16  4.16  3.97  4.06  4.13  3.79  4.26  4.44 |

^#^The cut-off value is 4.5 Å (includes van der Waals interaction).

All contacts are part of the N-terminal TM-JM helix.
