# Supplementary material for: Structural, mechanistic, and physiological insights into phospholipase A-mediated membrane phospholipid degradation in Pseudomonas aeruginosa
Source: eLife. 2022 May 10;11:e72824. doi: 10.7554/eLife.72824 (PMC9132575; doi:10.7554/eLife.72824)
Supplement: Supplementary file 7. [file elife-72824-supp7.docx]

**Supplementary File 7:** List of interactions^#^ involving the catalytic triad residues S137, D258 and H286.

| **Source atoms** | **Target atoms** | **Distance (Å)** |
| --- | --- | --- |
| *Monomer A*  Ser 137A N  Ser 137A O  conformation A  Ser 137 A OG  conformation B  Ser 137 A OG  Asp 258A N  Asp 258A OD1  Asp 258A OD2  His 286A N  His 286A ND1  His 286A NE2  His 286A O  *Monomer B*  Ser 137B N  Ser 137B O  conformation A  Ser 137B OG  conformation B  Ser 137B OG  Asp 258B N  Asp 258B OD1  Asp 258B OD2  His 286B N  His 286B ND1  His 286B NE2  His 286B O | Asn 136A ND2  Ile 160A O  Asp 161A O  Ala 163A N  Gly 139A N  Gly 140A N  His 141A N  His 286A NE2  HOH 229S O  MYR 500A O1  HOH 229S O  Gly 255A O  Leu 261A O  Leu 261A N  His 286A ND1  Arg 259A N  Val 260A N  Trp 254A NE1  Leu 261A O  Asp 161A OD2  His 286A ND1  Val 199A O  Asp 258A OD1  Asp 258A OD2  Ser 137A OG  Asn 136A ND2  Asn 136B ND2  Ile 160B O  Asp 161B O  Ala 163B N  Gly 140B N  His 141B N  Gly 139B N  His 286B NE2  HOH 171S O  IPA 504B C1<<<  HOH 171S O  Gly 255B O  Arg 259B N  Leu 261B N  Leu 261B O  Val 260B N  His 286B ND1  Trp 254B NE1  Asp 161B OD2  Leu 261B O  His 286B ND1  Val 199B O  Asp 258B OD2  Asp 258B OD1  Ser 137B OG  Asn 136B ND2 | 3.39  3.20  3.04  3.01  3.17  2.89  2.97  3.04  2.23  3.43  2.52  3.13  3.25  2.93  3.12  3.00  2.75  3.49  3.08  2.63  2.72  3.08  3.12  2.72  3.04  3.25  3.35  3.16  3.05  3.02  2.89  3.02  3.19  2.83  2.62  3.25  2.80  3.08  2.97  2.94  3.29  2.77  3.12  3.43  2.67  3.22  2.72  2.97  2.72  3.12  2.83  3.16 |

^#^The cut-off value for hydrogen bond selection used is ≤ 3.5 Å.
