# Supplementary material for: Structural, mechanistic, and physiological insights into phospholipase A-mediated membrane phospholipid degradation in Pseudomonas aeruginosa
Source: eLife. 2022 May 10;11:e72824. doi: 10.7554/eLife.72824 (PMC9132575; doi:10.7554/eLife.72824)
Supplement: Supplementary file 8. [file elife-72824-supp8.docx]

**Supplementary File 8:** Residues lining the active site cavity and their interactions with ligands.

| Residue | Interacting ligand |
| --- | --- |
| L27 | - |
| A28 | - |
| V30 | OG(A,B) |
| R31 | 11A |
| E34 | OG(A,B) |
| G70 | - |
| F71 | MYR, IPA503 |
| G72 | - |
| A73 | MYR |
| D74 | - |
| D76 | - |
| N77 | MYR, 11A |
| W78 | - |
| L79 | MYR, 11A |
| R80 | OG(A,B) |
| F81 | - |
| N136 | MYR, IPA503 |
| S137* | MYR, IPA503 |
| M138 | - |
| H141 | - |
| A163 | - |
| F174 | - |
| L173 | - |
| L184 | - |
| V185 | - |
| V186 | - |
| F192 | - |
| L195 | - |
| L196 | - |
| V199 | - |
| F200 | OG(A,B) |
| N203 | OG(A) |
| P204 | OG(A,B) |
| L206 | OG(A,B) |
| L210 | OG(B) |
| L214 | MYR |
| R217 | - |
| A218 | - |
| S222 | - |
| N225 | - |
| F229 | - |
| L232 | - |
| V260 | - |
| L261 | - |
| H286* | MYR, IPA503 |
| M289 | MYR |
| V287 | - |
| V290 | - |

*active site residues
