# Supplementary material for: Structural, mechanistic, and physiological insights into phospholipase A-mediated membrane phospholipid degradation in Pseudomonas aeruginosa
Source: eLife. 2022 May 10;11:e72824. doi: 10.7554/eLife.72824 (PMC9132575; doi:10.7554/eLife.72824)
Supplement: Supplementary file 9. [file elife-72824-supp9.docx]

**Supplementary File 9:** Michaelis-Menten constants for inhibition of PlaF with decanoic acid (FA C10).

| c(FA C10) [mM] | *K*_m_ [mM]* | *v*_max_ [*U*/mg]* |
| --- | --- | --- |
| 0.0 | 0.17±0.02 | 899.5±37.2 |
| 0.5 | 0.23±0.03 | 916.0±39.4 |
| 1.5 | 0.25±0.03 | 830.9±40.0 |
| 2.5 | 0.34±0.04 | 717.8±35.1 |
| 5.0 | 0.45±0.05 | 512.2±26.6 |
| 7.5 | 0.66±0.04 | 390.8±13.7 |

* Results are mean ± S.D. of three experiments each measured with three samples.
