# Supplementary material for: Structural, mechanistic, and physiological insights into phospholipase A-mediated membrane phospholipid degradation in Pseudomonas aeruginosa
Source: eLife. 2022 May 10;11:e72824. doi: 10.7554/eLife.72824 (PMC9132575; doi:10.7554/eLife.72824)
Supplement: Supplementary file 10. [file elife-72824-supp10.docx]

**Supplementary File 10:** Average 2D-RMSD_all atom_ of residues 25 to 315 of the structures sampled along MD trajectories.^[a]^

|  | di-PlaF_A_^[b]^ | di-PlaF_B_^[b]^ | PlaF_A_^[c]^ | t-PlaF_A_^[d]^ | PlaF_B_^[c]^ | t-PlaF_B_^[d]^ |
| --- | --- | --- | --- | --- | --- | --- |
| di-PlaF_A_^[b]^ | 3.42 ± 0.59 | 3.96 ± 0.69 | 3.63 ± 0.58 | 3.60 ± 0.61 | 3.94 ± 0.57 | 3.91 ± 0.60 |
| di-PlaF_B_^[b]^ |  | 4.01 ± 0.81 | 4.05 ± 0.68 | 4.05 ± 0.72 | 4.29 ± 0.70 | 4.23 ± 0.71 |
| PlaF_A_^[c]^ |  |  | 3.59 ± 0.60 | 3.71 ± 0.63 | 4.08 ± 0.58 | 4.02 ± 0.64 |
| t-PlaF_A_^[d]^ |  |  |  | 3.58 ± 0.72 | 4.05 ± 0.61 | 3.93 ± 0.65 |
| PlaF_B_ |  |  |  |  | 4.17 ± 0.76 | 4.21 ± 0.62 |
| t-PlaF_B_ |  |  |  |  |  | 3.99 ± 0.80 |

^[a]^ RSMD values in Å, mean ± S.D., were computed in a pair-wise manner for respective structures sampled every ns along the MD trajectories.

^[b]^ PlaF molecules in dimeric form starting from the crystal structure.

^[c]^ PlaF_A_ and PlaF_B_ obtained from the dimeric form by removal of the opposite chain.

^[d]^ PlaF_A_ and PlaF_B_ in the tilted monomeric form.
