# Supplementary material for: Structural, mechanistic, and physiological insights into phospholipase A-mediated membrane phospholipid degradation in Pseudomonas aeruginosa
Source: eLife. 2022 May 10;11:e72824. doi: 10.7554/eLife.72824 (PMC9132575; doi:10.7554/eLife.72824)
Supplement: Figure 5—source data 1. [file elife-72824-fig5-data1.pdf]

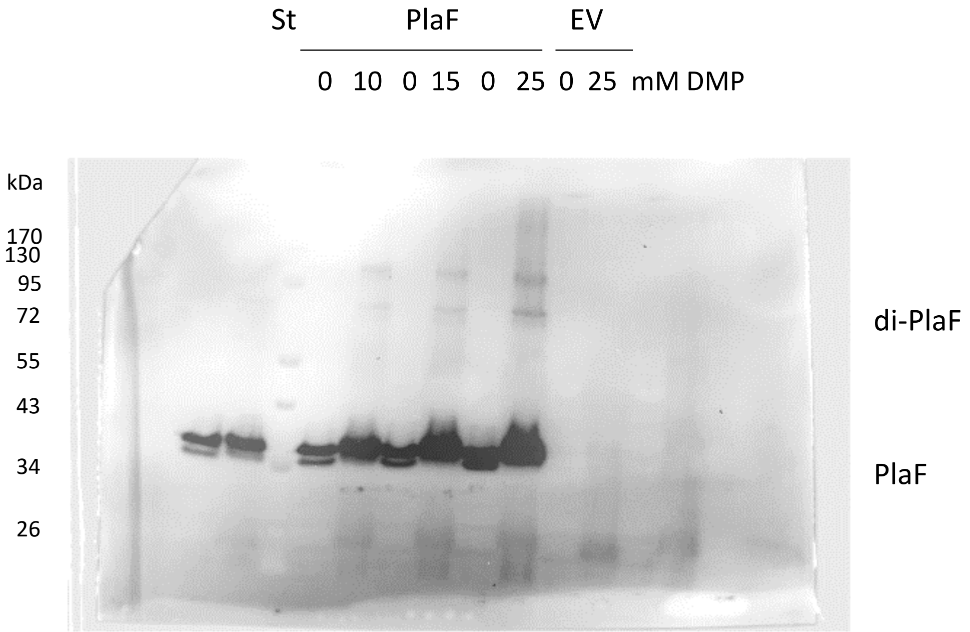

St
PlaF
EV
0 10 0 15 0 25 0 25 mM DMP
kDa
170
130
95
72
55
43
34
26
di-PlaF
PlaF

Figure 5a
