# Supplementary material for: Structural, mechanistic, and physiological insights into phospholipase A-mediated membrane phospholipid degradation in Pseudomonas aeruginosa
Source: eLife. 2022 May 10;11:e72824. doi: 10.7554/eLife.72824 (PMC9132575; doi:10.7554/eLife.72824)
Supplement: Transparent reporting form [file elife-72824-transrepform1.docx]

***eLife’s* transparent reporting form**

We encourage authors to provide detailed information *within their submission* to facilitate the interpretation and replication of experiments. Authors can upload supporting documentation to indicate the use of appropriate reporting guidelines for health-related research (see [EQUATOR Network](http://www.equator-network.org/%20)), life science research (see the [BioSharing Information Resource](https://biosharing.org/)), or the [ARRIVE guidelines](http://www.plosbiology.org/article/info:doi/10.1371/journal.pbio.1000412) for reporting work involving animal research. Where applicable, authors should refer to any relevant reporting standards documents in this form.

If you have any questions, please consult our Journal Policies and/or contact us: [editorial@elifesciences.org](mailto:editorial@elifesciences.org).

**Sample-size estimation**

- You should state whether an appropriate sample size was computed when the study was being designed
- You should state the statistical method of sample size computation and any required assumptions
- If no explicit power analysis was used, you should describe how you decided what sample (replicate) size (number) to use

Please outline where this information can be found within the submission (e.g., sections or figure legends), or explain why this information doesn’t apply to your submission:

We have used the same sample size as usually reported for similar experiments published in other articles.

**Replicates**

- You should report how often each experiment was performed
- You should include a definition of biological versus technical replication
- The data obtained should be provided and sufficient information should be provided to indicate the number of independent biological and/or technical replicates
- If you encountered any outliers, you should describe how these were handled
- Criteria for exclusion/inclusion of data should be clearly stated
- High-throughput sequence data should be uploaded before submission, with a private link for reviewers provided (these are available from both GEO and ArrayExpress)

Please outline where this information can be found within the submission (e.g., sections or figure legends), or explain why this information doesn’t apply to your submission:

Biological replicates are samples obtained from cultures grown from a single colony from the Agar plate. When one sample was measured several times we refer to it as a technical replicate.

Enzyme activities and other biochemical studies were usually measured in three independent experiments and at least 3 technical replicates were used per experiment. Usually, results were reproduced using two batches of protein purification. This is applicable for figures: 1a, 1c, 2a, 2b, 5, 6a, 6b. This information is available in the figure captions.

SDS-PAGE and Western blot analysis were performed at least 2 times and representative pictures were shown.

In lipidomics and virulence experiments 3-4 biological replicates were used. This is applicable for figures 2c and 3. This information is available in the figure captions.

In unbiased computational simulations, results from 10 replicas for each protein starting structure were used. Sampling information used for PMF calculations was split into 4 independent fractions of 50 ns length in each case. This information is available in the figure captions. This is applicable for figure 7 and figure 7-supplementary figure 1.

In lipidomics analysis one sample of *P. aeruginosa* Δ*plaF*::*plaF* contained too few phospholipids after extraction with organic solvent; therefore, this sample was not analysed by MS. Elsewhere, no outliers were obtained.

**Statistical reporting**

- Statistical analysis methods should be described and justified
- Raw data should be presented in figures whenever informative to do so (typically when N per group is less than 10)
- For each experiment, you should identify the statistical tests used, exact values of N, definitions of center, methods of multiple test correction, and dispersion and precision measures (e.g., mean, median, SD, SEM, confidence intervals; and, for the major substantive results, a measure of effect size (e.g., Pearson's r, Cohen's d)
- Report exact p-values wherever possible alongside the summary statistics and 95% confidence intervals. These should be reported for all key questions and not only when the p-value is less than 0.05.

Please outline where this information can be found within the submission (e.g., sections or figure legends), or explain why this information doesn’t apply to your submission:

Information about used statistical methods is provided in the caption of each figure where applicable.

(For large datasets, or papers with a very large number of statistical tests, you may upload a single table file with tests, Ns, etc., with reference to sections in the manuscript.)

**Group allocation**

- Indicate how samples were allocated into experimental groups (in the case of clinical studies, please specify allocation to treatment method); if randomization was used, please also state if restricted randomization was applied
- Indicate if masking was used during group allocation, data collection and/or data analysis

Please outline where this information can be found within the submission (e.g., sections or figure legends), or explain why this information doesn’t apply to your submission:

Not applicable.

**Additional data files (“source data”)**

- We encourage you to upload relevant additional data files, such as numerical data that are represented as a graph in a figure, or as a summary table
- Where provided, these should be in the most useful format, and they can be uploaded as “Source data” files linked to a main figure or table
- Include model definition files including the full list of parameters used
- Include code used for data analysis (e.g., R, MatLab)
- Avoid stating that data files are “available upon request”

Please indicate the figures or tables for which source data files have been provided:

Source data were provided for the following figures in the main text and in supporting material:

Figure 1a-source data 1: Uncropped Western blot shown in figure 1a.

Figure 1b-source data 1: Uncropped Western blot shown in figure 1b.

Figure 1c-source data 1: Uncropped Western blot shown in figure 1c.

Figure 1d-source data 1: Uncropped Western blot shown in figure 1d.

Source data-Figure 1a: Excel file with data used to make figure 1a.

Source data-Figure 1c: Excel file with data used to make figure 1c.

Source data-Figure 2a: Excel file with data used to make figure 2a.

Source data-Figure 2b: Excel file with data used to make figure 2b.

Figure 5a-source data 1: Uncropped Western blot shown in figure 5a.

Figure 5b-source data 1: Uncropped SDS-PAGE shown in figure 5b.

Figure 5c-source data: Origin file with data used to make figure 5c.

Source data-Figure 6a: Excel file with data used to make figure 6a.

Figure 6c-source data 1: Uncropped SDS-PAGE shown in figure 6c.

Source data-Figure 6a: Excel file with data used to make figure 6a.

Source data-Figure 6b: Excel file with data used to make figure 6b.

Source data-Figure 2-figure supplement1: Excel file with data used to make Figure 2-figure supplement 1.

Source data-Figure 3-figure supplement 1: Excel file with data used to make Figure 3-figure supplement 1.

Figure 5-figure supplement 1 -source data 1: Uncropped SDS-PAGE shown in figure S10.

Figure 5-figure supplement 1 -source data 2: Original file of the SDS-PAGE shown in Figure 5-figure supplement 1.

Source data- Figure 5-figure supplement 2: Excel file with data used to make Figure 5-figure supplement 2.

Figure 7-figure supplement 2 -source data 1: Uncropped Western blot shown in Figure 7-figure supplement 2.

Source data used to calculate the potentials of mean force and their corresponding simulation trajectory files shown in figure 7 and figure 7-supplementary figure 1 are accessible at the DSpace instance researchdata.hhu.de under DOI: 10.25838/d5p-31.
